# Restriction endonuclease selective inhibition by β-cyclodextrin sulfate

**DOI:** 10.1042/BSR20250403

**Published:** 2025-02-18

**Authors:** Bekir C. Celikkaya, Fraser J. Scott

**Affiliations:** Department of Pure and Applied Chemistry, Faculty of Science, University of Strathclyde, Thomas Graham Building, 295 Cathedral Street, G1 1XL Glasgow, U.K.

**Keywords:** β-Cyclodextrin sulfate, DNA, restriction endonucleases

## Abstract

Restriction endonucleases (REs) are sequence-specific DNA-cleaving enzymes that are widely used as tools in molecular biology. Although REs is not typically considered therapeutic targets, their inhibition provides a useful framework for understanding how DNA-binding enzymes can be differentially modulated. In this present study, we investigated the inhibitory activity of β-cyclodextrin sulfate (β-CDsul), a highly anionic macrocycle previously reported to act as a non-selective inhibitor of restriction enzymes. Using NdeI-linearised pBR322 as a defined substrate, we quantified the inhibition of EcoRI, HindIII, and VspI under optimised digestion conditions. β-CDsul inhibited EcoRI with substantially greater potency than HindIII or VspI, exhibiting a 13- to 27-fold difference in IC_50_ values. In contrast, EDTA inhibited all three enzymes with comparable potency, consistent with non-selective divalent metal ion chelation. Selective inhibition of EcoRI by β-CDsul was maintained in a dual-enzyme system containing both EcoRI and VspI, supporting an enzyme-dependent mode of action. These findings demonstrate that β-cyclodextrin sulfate acts as a selective inhibitor of EcoRI and highlight the potential of polyanionic macrocycles as biochemical probes for differential endonuclease activity.

## Background

DNA-binding proteins have diverse roles in replication, transcription, repair, and degradation of DNA [1,2]. Among these, DNA restriction endonucleases (REs) are prokaryotic enzymes that cleave double-stranded DNA within or near specific recognition sites [3]. Typically, they require divalent cations, such as Mg^2+^, for activity [4]. They are thought to protect host cells from invading foreign DNA, such as bacteriophage genomes. Type II REs, in particular, have been widely studied and have become indispensable tools in molecular cloning [5–7]. Structural studies of type II REs have shown that DNA recognition often involves a combination of base-specific contacts, interactions with the phosphate backbone, and conformational changes upon substrate engagement, highlighting the complex mechanisms underlying sequence-specific cleavage [8].

Although REs themselves are not typical therapeutic targets, they serve as useful models for understanding how DNA-binding enzymes can be selectively inhibited [9,10]. For example, SELEX-derived RNA aptamers have been developed that can selectively inhibit KpnI over BamHI or PacI, with IC_50_ (half maximal (50%) inhibitory concentration)s of 20–150 nM [11]. Beyond bacterial REs, small-molecule inhibitors have been developed against other endonucleases, including the influenza PA endonuclease, with clinically approved drugs such as baloxavir marboxil illustrating that nuclease inhibition can be an effective antiviral strategy [12,13].

Cyclodextrins are known to have considerable uses within the pharmaceutical industry, particularly within formulation science, but are less well known for direct biological activity [14–16]. Tauran et al. showed that β-cyclodextrin sulfate (β-CDsul), a highly anionic macrocycle, was able to inhibit REs with limited selectivity. Specifically, Nrul and HindIII bacterial REs were both inhibited by β-CDsul (Nrul IC_50_ = 3 μM and HindIII IC_50_ = 6 μM) against digestion of λ DNA. Interestingly, no inhibition was observed by treatment with the uncharged β-CD, indicating that the anionic sulphate groups are crucial for inhibitory activity [17]. A presumed hypothesis for the non-selective action of β-CDsul relates to the sequestration of Mg^2+^ from the active sites of REs due to its high negative charge density or favourable interactions with the highly positively charged regions of REs necessary for binding to polyanionic DNA.

As part of a different research programme, looking at sequence-specific interactions between small molecules and DNA, we required a non-specific inhibitor of REs to act as a control, and consequently we selected β-CDsul [18]. However, we observed that β-CDsul had different inhibitory activities on different REs, making it an unsuitable control, but prompted the present study.

## Hypothesis

We hypothesise that β-CDsul is a selective inhibitor of restriction endonuclease. In particular, it is selectively able to inhibit the action of EcoRI, over HindIII and VspI, against Ndel-linearised pBR322 as a model DNA substrate.

## Materials and methods

### pBR322 linearization

pBR322 (SD0041, Thermo Fisher Scientific™ Baltics UAB (isolated from *Escherichia coli* (dam+, dcm+))), Ndel enzyme (ER0582, Thermo Fisher Scientific™ Baltics UAB (sourced from *Neisseria denitrificans*)) and Buffer O (Supplementary Table S1) were purchased from ThermoFisher Scientific^®^. To linearise pBR322, 0.5 μg in 1 μl was digested with 1 μl (1.25 U/μl) of Ndel, 2 μl of Buffer O, and 16 μl of molecular biology grade water by incubating at 37°C for 2 h (Supplementary Figure S1). After digestion, Ndel was inactivated by heating to 65°C for 20 min.

### Restriction endonuclease inhibition assay

EcoRI, HindIII, VspI, Buffer O, Buffer EcoRI, and Buffer R were purchased from Thermo Fisher Scientific^®^ (Thermo-Fischer Scientific™, ER0271, ER0501, ER0911).

Digestion time course studies were conducted for each RE by incubating 2 μl of NdeI-linearised pBR322 (2.5 ng/μl), 1 μl of RE (1.25 U/μl), 2 μl of appropriate buffer, and 15 μl of molecular biology grade water at 37°C for various times (Supplementary Figure S1). The minimum time for 100% digestion by each RE was determined to be 20 min for EcoRI and VspI and 40 min for HindIII. These times were used as the digestion time for the RE inhibition assays.

To determine the effects of β-CDsul treatment on REs, Nl-pBR322 was incubated at 37°C with EcoRI, VspI, or HindIII for the previously determined amount of time.

β-CDsul and EDTA stock solutions were made in molecular biology grade water, and dilution series were prepared at appropriate concentrations to give the necessary final concentrations of each in the assay mixture. The assay mixture composition was 2 μl of Nl-pBR322 (2.5 ng/μl), 1 μl of RE (1.25 U/μl), 2 μl of appropriate buffer, 1 μl of β-CDsul or EDTA at desired concentration, and 14 μl of molecular biology grade water. As a negative control, 1 μl extra of microbiology grade water was used instead of EDTA or β-CDsul. After the appropriate amount of incubation time at 37°C, the REs were thermally inactivated by incubation at 65°C for 20 min.

For co-digestion assays with EcoRI and VspI, the conditions were as follows: 2 μl of Nl-pBR322 (2.5 ng/μl), 1 μl of each RE (1.25 U/μl), 5 μl of EcoRI buffer, 2 μl of VspI buffer (buffer O), 1 μl of β-CDsul at the desired concentration, and 38 μl of molecular biology grade water. The digestion time was either variable or fixed at 120 min for the variable concentration experiment to achieve full digestion. The composition of the components with the co-digestion assays was more complex than single digestion systems due to the need for simultaneously optimising the activity of each RE in the non-ideal buffer system of the other RE. It should therefore be noted that the rate of digestion of each RE in the co-digestion system is different from their rates in the single digestion system.

### Agarose gel electrophoresis

Electrophoresis gel solution was prepared using 0.8% (w/v) agarose (Thermo Scientific™ Agarose I (Molecular Biology Grade)) with 1× TBE Buffer (Thermo Scientific™ TBE Buffer (Tris–borate–EDTA) (10×)). The mixture was heated until the agarose was completely dissolved and the solution became clear. Sybr Safe DNA stain (1% (v/v), Invitrogen™ SYBR™ Safe DNA Gel Stain) was added into the agarose solution. The final solution was poured into the gel tray and cooled down to room temperature (25°C). Ten microliters of samples from digestion experiments was mixed well with 1 μl of 500× diluted SYBR Safe Gel Stain (ThermoFisher Scientific™ Invitrogen, catalogue number S33102) and 1 μl of 5× diluted FlashGel Loading Dye (Lonza Bioscience, catalogue number 50462) and then loaded into wells. A FlashGel™ Dock system was used for electrophoresis with a voltage of 0–300 VDC, power of 15 W, and current of 50 mA.

Gel images were captured using a UV-transilluminator capturing system (Syngene™ Ingenius 3 Manual Gel Documentation System) with the following parameters: resolution: 3.0 MP, bit depth: 12/16 bit, and wavelength: 302 nm. The images were analysed using GelAnalyzer [19] software by calculating the normalised intensities of fragment bands.

## Results

**Figure 1:**
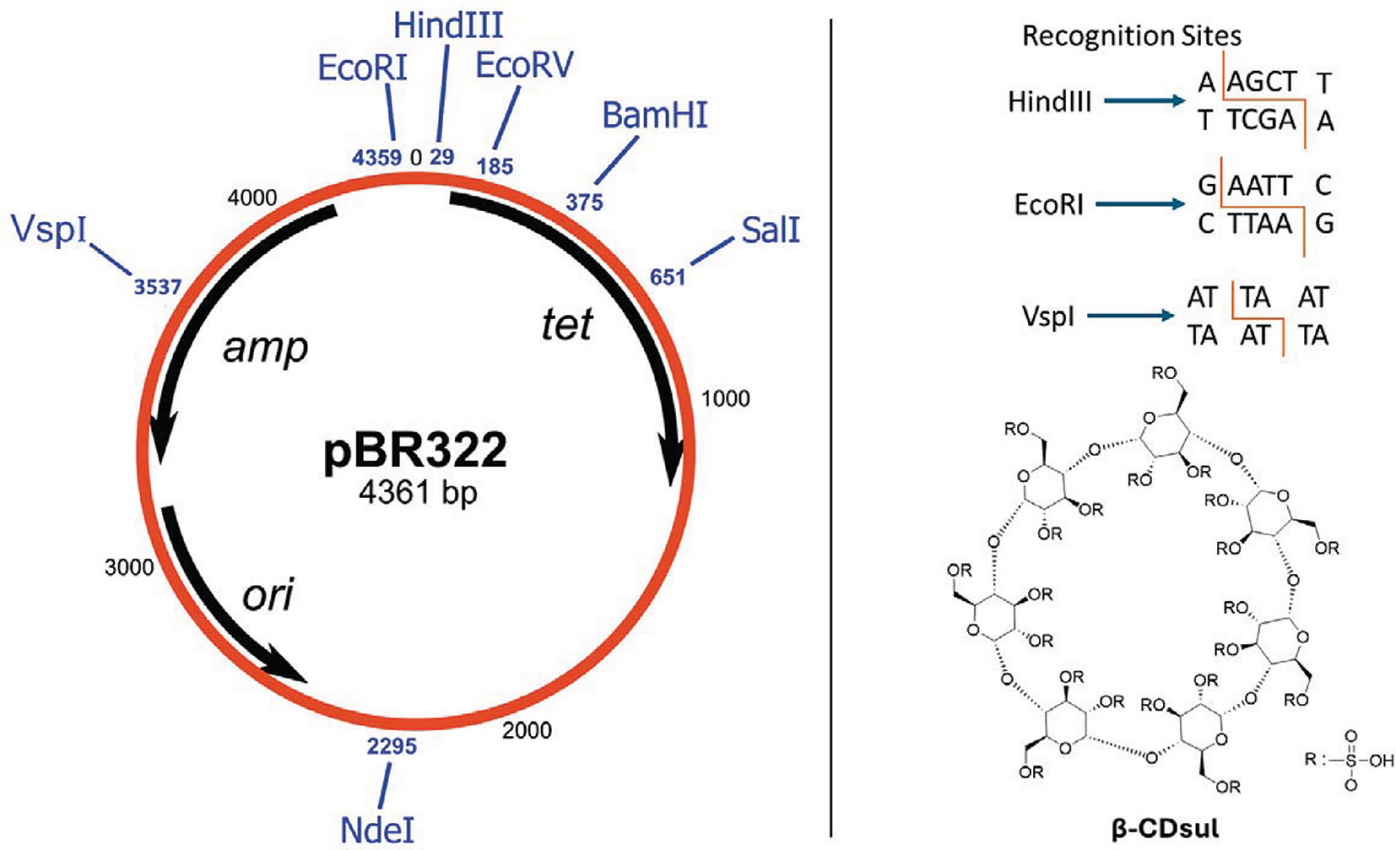
pBR322 and β-CDsul. Restriction enzymes cutting locations on pBR322 plasmid and their recognition sites with the chemical structure of β-CDsul.

We compared the inhibitory activity of β-CDsul against REs with different restriction sequences. Specifically, EcoRI, which cuts at 5′-G/AATTC-3′; HindIII, which cuts at 5′-A/AGCTT-3′; and VspI, which cuts at 5′-AT/TAAT-3′ sites (Figure 1). Our chosen substrate was pBR322, which has only one restriction site for these enzymes and thus would give a simple-to-interpret band profile on subsequent analysis by gel electrophoresis. However, the electrophoretic migration rate of circular and linearised pBR322 is very similar, so we elected to use linearised pBR322 as the starting substrate for restriction inhibition experiments. To linearise, we digested with Ndel, as its restriction site (2295 bp) is far away from the restriction sites of EcoRI (4359 bp), HindIII (29 bp), and VspI (3537 bp), ensuring the formation of two digested fragments that are large and easy to observe (Figure 1).

Therefore, Nl-pBR322 was digested with either EcoRI, HindIII, or VspI, and we investigated their relative digestion rates by monitoring with gel electrophoresis. We found that to obtain 100% digestion, EcoRI and VspI required 20 min of incubation; however, HindIII required 40 min of incubation (Supplementary Figure S1). Given the different digestion rates, and to ensure the effects of inhibitors could be fairly compared between different REs, the protocol for follow-on IC_50_ determination used RE-specific digestion times corresponding to the time to achieve 100% digestion.

**Figure 2:**
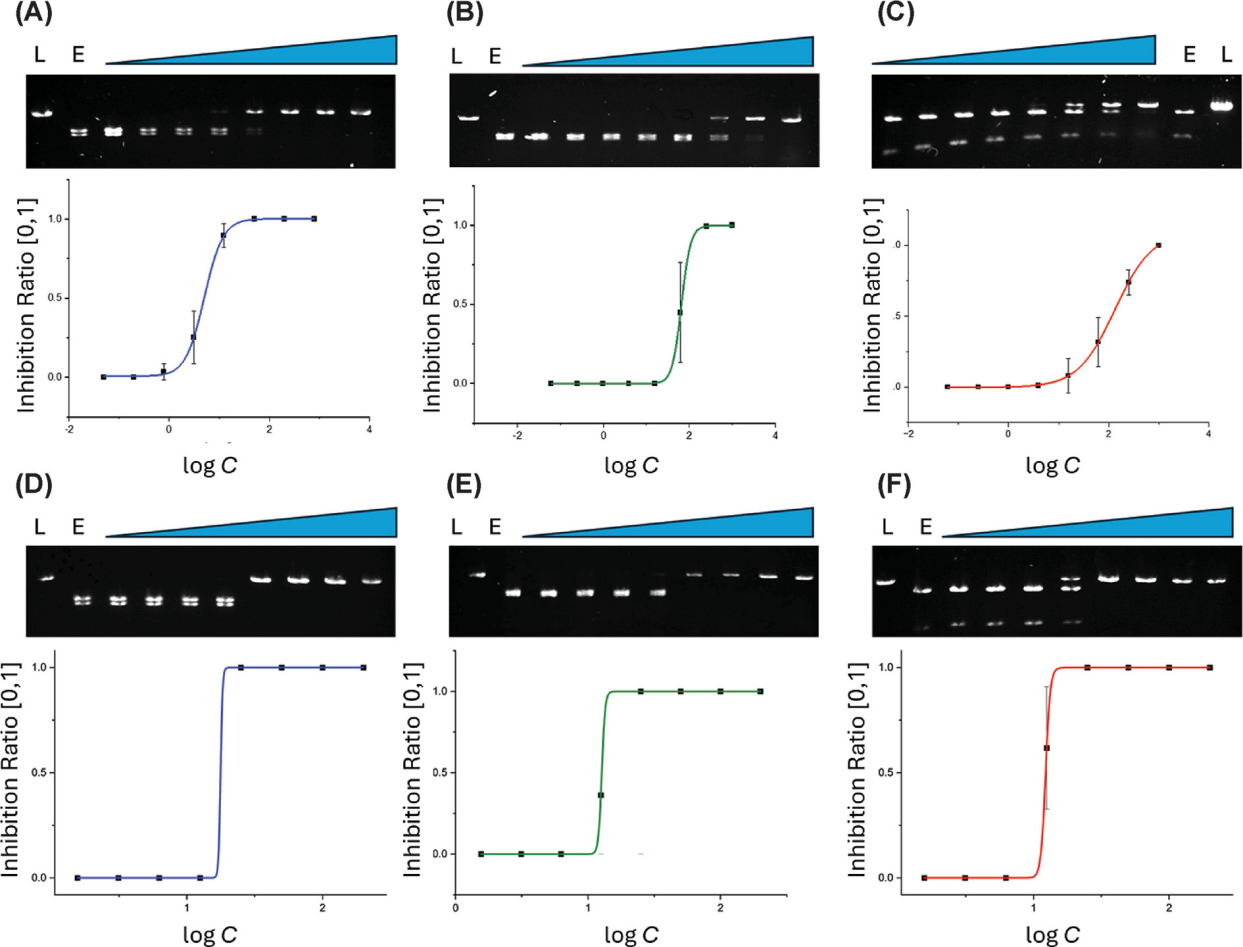
β-CDsul and EDTA inhibition of REs. Agarose gel images and IC_50_ graphs for (**A**) EcoRI+β-CDsul (0.04–800 μM), (**B**) HindIII+β-CDsul (0.06–1000 μM), (**C**) VspI+β-CDsul (0.06–1000 μM), (**D**) EcoRI + EDTA (1.56–200 μM), (**E**) HindIII + EDTA (1.56–200 μM), and (**F**) VspI + EDTA (1.56–200 μM) digested plasmid fragments (L: Nl-pBR32 linear control (DNA only); E: Enzyme-digested Nl-pBR322 (without ligand); blue triangle; increased concentration of ligands).

With an optimised assay protocol, we next obtained the inhibition IC_50_s of β-CDsul against each RE of interest (Figure 2). We are not aware of any non-selective inhibitors of REs reported in the literature, and therefore we elected to use EDTA as a control, hypothesising that its chelating properties would non-selectively inhibit REs by sequestering crucial Mg^2+^ ions from the active site of the REs (Figure 2) [4,20].

As intended, EDTA is a relatively non-selective inhibitor across all three REs, with IC_50_s of 12, 13, and 18 μM against VspI, HindIII, and EcoRI, respectively. It is a little less effective against EcoRI, but the difference in IC_50_s is negligible in comparison with what is observed for β-CDsul, giving some confidence that IC_50_ differences are not due to different conditions used to determine the IC_50_s for each RE. The IC_50_s of β-CDsul against VspI, HindIII, and EcoRI are 133, 66, and 5 μM, respectively. There is only about a two-fold difference in IC_50_ of β-CDsul against VspI and HindIII; however, β-CDsul is about 27-fold more potent at inhibiting EcoRI compared with VspI and 13-fold more for EcoRI compared with HindIII. Taken together, these data suggest that β-CDsul is a selective inhibitor of EcoRI over HindIII or VspI.

Having determined that β-CDsul is a selective inhibitor of EcoRI, over both HindIII and VspI, in single RE systems, we were next interested in exploring a more complex dual RE system (Figure 4A,B). We therefore monitored the digestion of Nl-pBR322 by both EcoRI and VspI over time in the presence (10 μM (2× IC_50_)) and absence of β-CDsul (Supplementary Figure S2A and Figure 4C). As an alternative investigation, we also monitored the extent of digestion in the same system at a single time point (120 min), but in the presence of different concentrations of β-CDsul (Supplementary Figure S2B and Figure 4D).

**Figure 3:**
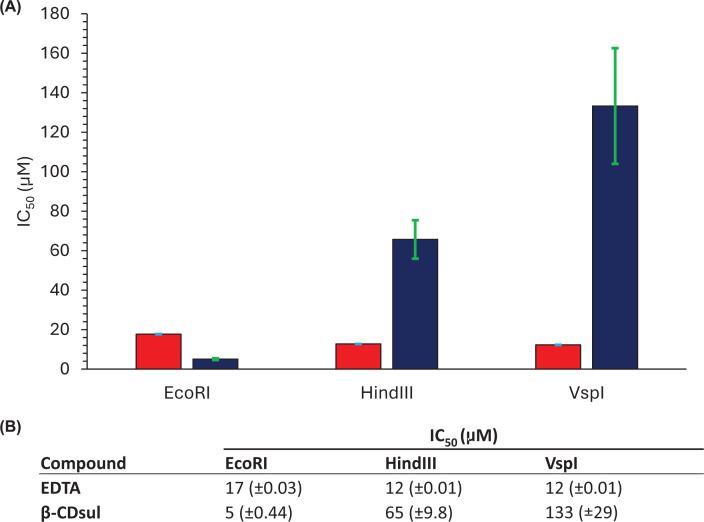
IC_50_s of β-CDsul and EDTA inhibition of REs. The IC_50_ bar chart (**A**) and table (**B**) of EcoRI, HindIII, and VspI enzyme digestion with β-CDsul and EDTA treatment to Nl-pBR322 plasmid DNA.

The dual RE time course study shows complex kinetics in both the untreated and treated systems, due to two pathways to generate some of the pBR322 fragments and different digestion rates for each fragment (Figure 3A,B). Indeed, it would appear that in this dual RE system that the rate of digestion of EcoRI is considerably slower than VspI, evidenced by no observation of fragment D, likely due to competition between the two REs rather than non-ideal enzyme buffers (Supplementary Table S1). Nonetheless, there is clear evidence of selective inhibition of EcoRI by β-CDsul. For example, tracking the concentration of fragment A over time shows a progressive decrease in its presence in the untreated group corresponding to the action of EcoRI after its rapid formation by initial digestion by VspI (prior to first measurement at 30 min). However, in the β-CDsul-treated system, following a slightly inhibited delay in the formation of fragment A (maximum concentration at 60 min), its concentration does not decrease until 240 min. Indeed, at the 180 and 210 min time points, there is no evidence of fragment A in the β-CDsul-treated system, compared with the maximum concentration in the untreated system.

**Figure 4:**
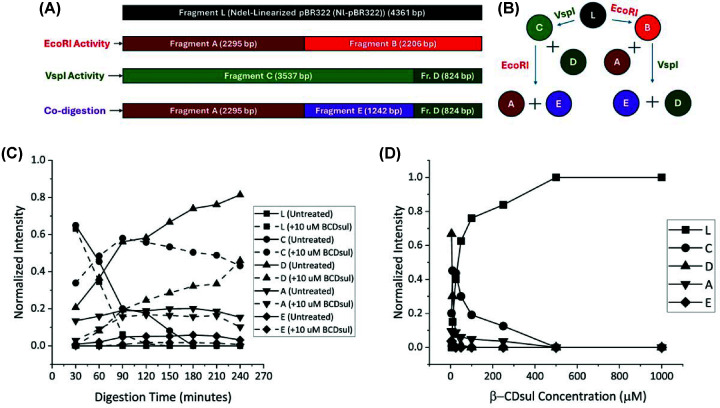
β-CDsul inhibition of RE co-digestion. (**A**) The cutting sites of EcoRI and VspI enzymes on Nl-pBR322, showing all potential fragments after the complete co-digestion. (**B**) A fraction map of Nl-pBR322 for illustrating the consecutive cuts by EcoRI and VspI, with the intermediate fragments. (**C**) Normalised band intensity of fragments for untreated and treated (10 μM β-CDsul) EcoRI/VspI co-digestion over time (30–240 min). Separate graphs for each band are available in Supplementary Figure S3. (**D**) Normalised band intensity of fragments for a range of β-CDsul concentrations (5–1000 μM) against EcoRI/VspI co-digestion.

For the β-CDsul concentration-dependent experiment, we again see complexity due to multiple pathways towards some of the fragments. Five hundred micromolar of β-CDsul results in no digestion by either RE. At the lower concentrations, we can see that there is a significant increase in EcoRI inhibition between 5 and 10 μM, illustrated in the increased concentration of fragment A as the concentration of β-CDsul increases, but still no Nl-pBR322, indicating VspI is less affected.

## Discussion

This work arose from the practical need for a non-specific inhibitor of restriction endonuclease activity to act as a control in studies examining sequence-specific interactions between small molecules and DNA. β-CDsul was selected based on prior reports that sulphated cyclodextrins can inhibit restriction endonuclease activity in a non-selective manner. However, systematic evaluation revealed pronounced differences in inhibitory potency between enzymes, prompting a detailed investigation of its effects on EcoRI, HindIII, and VspI. By using NdeI-linearised pBR322 as a defined substrate and enzyme-specific digestion times to normalise assay conditions, we were able to directly compare inhibition across enzymes and benchmark these effects against EDTA as a non-selective control.

Quantitative IC_50_ measurements demonstrate that β-CDsul inhibits EcoRI (5 μM) substantially more potently than HindIII (65 μM) or VspI (133 μM), with a 13- to 27-fold difference in inhibitory activity. In contrast, EDTA inhibited all three enzymes with comparable IC_50_ values, consistent with its likely mechanism of action through divalent metal ion chelation rather than enzyme-specific interactions. The narrow range of EDTA IC_50_ values (12–17 μM) supports the conclusion that the observed selectivity of β-CDsul does not arise from differences in digestion kinetics, buffer composition, or assay sensitivity. Instead, these data indicate that β-CDsul engages individual restriction endonucleases in a manner that results in differential functional inhibition.

The selective inhibition of EcoRI by β-CDsul was also evident in a dual-enzyme digestion system containing both EcoRI and VspI, where inhibition of EcoRI activity persisted despite competition for substrate. Although interpretation of such systems is complicated by parallel reaction pathways and enzyme competition, the persistence of EcoRI inhibition under these conditions further supports the conclusion that β-CDsul acts in an enzyme-selective manner rather than simply suppressing restriction activity globally.

A notable difference exists between the HindIII IC_50_ values reported previously, 6 μM (in reference [17]), and the 65 μM measured in the present study. It is well recognised that apparent IC_50_ values are dependent on experimental design, including substrate concentration, enzyme concentration, and the timing of measurements; for example, measured IC_50_ values can vary with changes in substrate levels relative to Km and with assay incubation time, because these factors influence the competitive dynamics between inhibitor and substrate and the extent of inhibition detected under end-point conditions [21]. Indeed, their studies used λ DNA at an unknown concentration, a ten-fold higher mass of DNA, and a longer incubation time. Secondly, buffer composition and ionic strength substantially influence restriction enzyme activity and inhibitor binding, particularly for polyanionic ligands that interact electrostatically with protein surfaces [22]. Here, we both used different buffer systems. Taken together, these variables can account for the order-of-magnitude difference in IC_50_ measurements.

Selective modulation of restriction endonuclease activity by supramolecular scaffolds has precedent in the literature. Carvalho *et al.* demonstrated that cucurbituril macrocycles inhibit DNA cleavage by multiple type II REs and that inhibition can be reversed by competitive binding agents, indicating that these effects arise from non-covalent interactions rather than irreversible active-site disruption [23]. Their experiments found that the cucurbituril CB7 inhibited KpnI, SacI, and XapI with IC_50_s of about 90, 250, and 500 μM, respectively, against plasmid pGL3-Basic DNA. Slightly elevated IC_50_s of about 200, 350, and 500 μM were obtained for the same REs, respectively, against linearised pGL3-Basic DNA. While the IC_50_s of β-CDsul compare favourably, it should be noted that the different assay conditions preclude a direct comparison. While β-CDsul differs chemically from cucurbiturils, both represent highly charged, water-soluble macrocycles capable of interacting with protein surfaces. More broadly, sulphated carbohydrates are known to interact with proteins through electrostatic complementarity, particularly at regions of high positive charge density, as extensively documented for heparin–protein interactions [24,25].

Additionally, SELEX-derived RNA aptamers have been shown to inhibit specific REs competitively, with high nanomolar to low micromolar affinities across different REs [11]. Most notably, several aptamers had IC_50_s of 13–36 nM for KpnI inhibition, while having no inhibitory activity at 40 μM for inhibition of BamHI or PacI. Although a direct comparison between these IC_50_s and those of β-CDsul cannot be made due to differences in assay conditions, it is notable that these RNA aptamers have molecular weights roughly an order of magnitude higher than β-CDsul.

A plausible interpretation of our data is that β-CDsul interacts with positively charged regions involved in DNA engagement or structural stabilisation, and that differences in surface charge distribution, electrostatic potential, or conformational flexibility between restriction endonucleases result in differential susceptibility to inhibition. Although type II restriction endonucleases share a common catalytic architecture, including the PD-(D/E)XK motif, structural studies have shown substantial variation in domain organisation, DNA-binding topology, and electrostatic landscapes surrounding the DNA-binding interface [26]. These variations may influence how polyanionic molecules approach or transiently associate with the enzyme, leading to selective perturbation of activity without requiring fundamentally different recognition mechanisms.

Importantly, this behaviour distinguishes β-CDsul from general chelators such as EDTA and highlights its potential utility as a biochemical probe for differential endonuclease activity. It suggests that polyanionic macrocycles can differentially modulate closely related endonucleases, providing a potential route to biochemical probes capable of dissecting enzyme-specific contributions in complex reaction systems or further interest in such molecules as therapeutics.

## Future directions

Several avenues for further investigation arise from this work. Firstly, structural and biophysical studies would be valuable in identifying the molecular determinants of β-CDsul selectivity, particularly noting that crystal structures are available for EcoRI and HindIII, but not VspI. Secondly, expanding the enzyme panel to include additional type II restriction endonucleases from different structural subfamilies and exploring more combinations would establish whether EcoRI-selective inhibition represents a broader trend or a more isolated phenomenon. Thirdly, systematic variation of cyclodextrin substitution patterns, particularly sulfate density and spatial arrangement, could clarify structure–activity relationships and determine whether selectivity can be tuned rationally. Finally, kinetic analyses examining effects on binding versus cleavage steps would help disentangle whether β-CDsul primarily interferes with substrate association or catalytic turnover during the restriction process.

## Supplementary Material

Supplementary Figures S1-S3 and Table S1

## Data Availability

The data supporting this article have been included in the ESI.

## Abbreviations

β-CDsul: β-cyclodextrin sulfate
REs: restriction endonucleases
