# Supplementary material for: Restriction endonuclease selective inhibition by β-cyclodextrin sulfate
Source: Biosci Rep. 2025 Feb 18;46(2):BSR20250403. doi: 10.1042/BSR20250403 (PMC13071375; doi:10.1042/BSR20250403)
Supplement: Supplementary Figures S1-S3 and Table S1 [file BSR-2025-0403_supp.pdf]

Restriction Endonuclease Selective Inhibition of β-Cyclodextrin Sulfate

Bekir C. Celikkaya and Fraser J. Scott*

Department of Pure and Applied Chemistry, Faculty of Science, University of Strathclyde, Thomas Graham Building, 295 Cathedral Street, G1 1XL, Glasgow, United Kingdom

*E-mail(s): fraser.j.scott@strath.ac.uk

Supplementary Information

Contents

1. Buffer Composition **Page 2**

2. RE Digestion Time Optimisation **Page 3**

3. EcoRI and VspI Co-digestion **Page 4**

1. Buffer Composition

Table S1. Chemical composition and pH values of endonuclease enzyme buffers[†]

Enzyme	Buffer Name	pH	Tris-HCI (mM)	$MgCI_2$ (mM)	BSA[*] (mg/mL)	NaCl (mM)	KCI (mM)	Triton X-100 (%)
NdeI, VspI	O	7.5	50	10	0.1	100	-	-
EcoRI	EcoRI	7.5	50	10	0.1	100	-	0.02
HindIII	R	8.5	10	10	0.1	-	100	-

**BSA=Bovine Serum Albumin*

†ThermoFisher Scientific ® product manuscripts

2. RE Digestion Time Optimisation

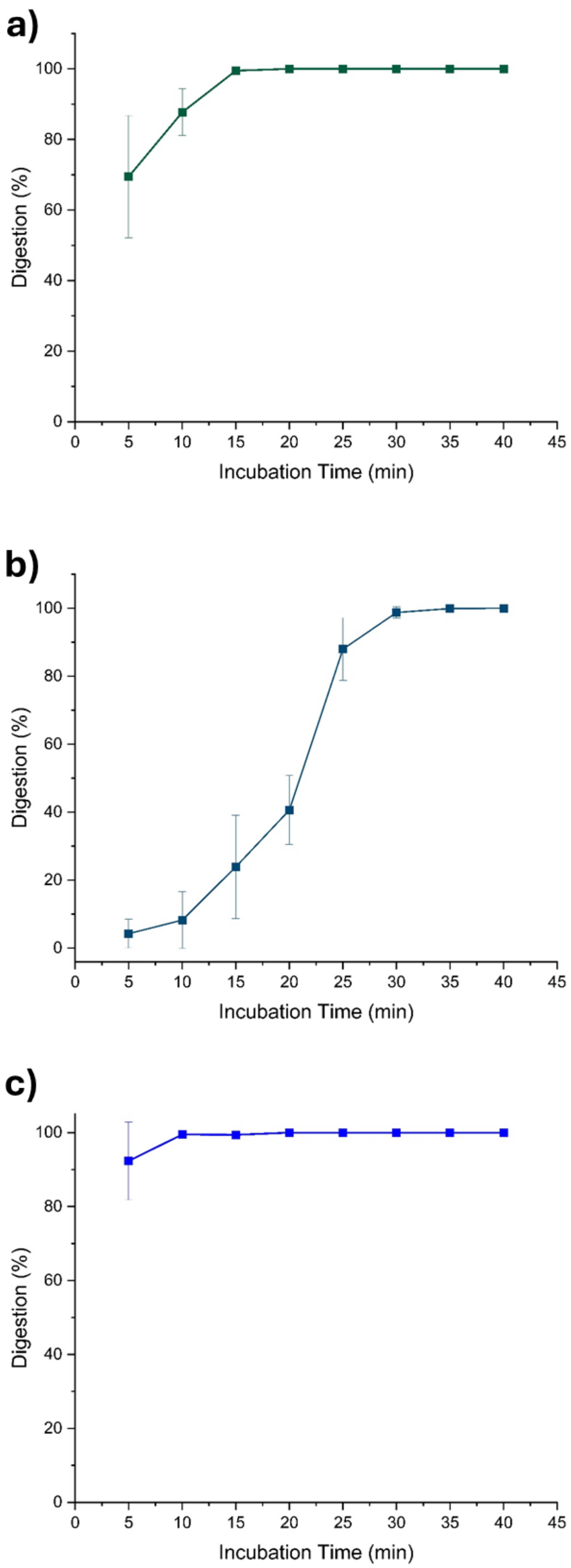

Figure S1: Time-dependent digestion profile of pBR322 by EcoRI (a), HindIII (b) and VspI (c). The percentage of digestion is plotted as a function of incubation time (minutes).

3. EcoRI and VspI Co-digestion

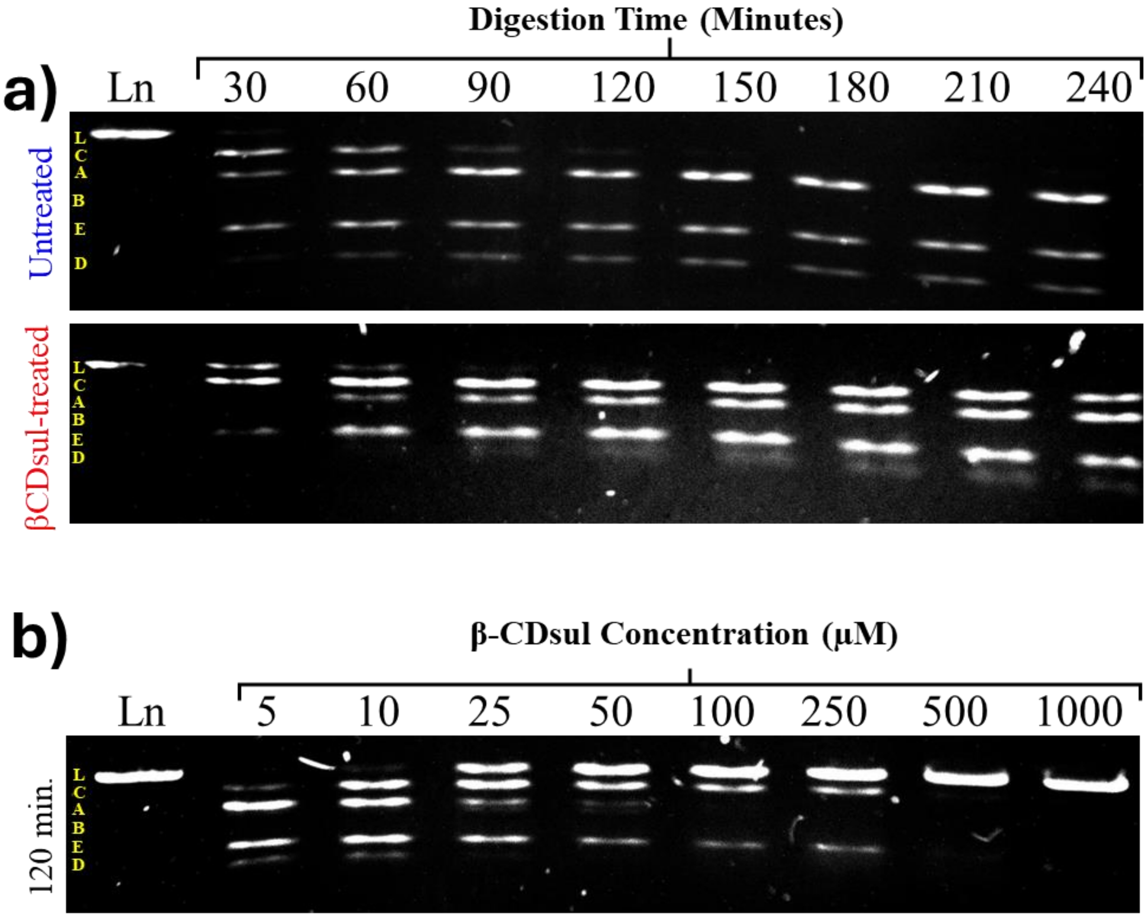

Figure S2: (a) Gel images for variable digestion times (30-240 minutes) of Nl-pBR322 by EcoRI/VspI co-digestion as untreated and treated with 10 μM β-CDsul. (b) The EcoRI/VspI co-digestion at 120 minutes digestion with a range of (5-1000 μM) β-CDsul concentrations. The "Ln" indicates the Nl-pBR322 linear DNA control, and letters in yellow are correspondingly show the fragments' position.

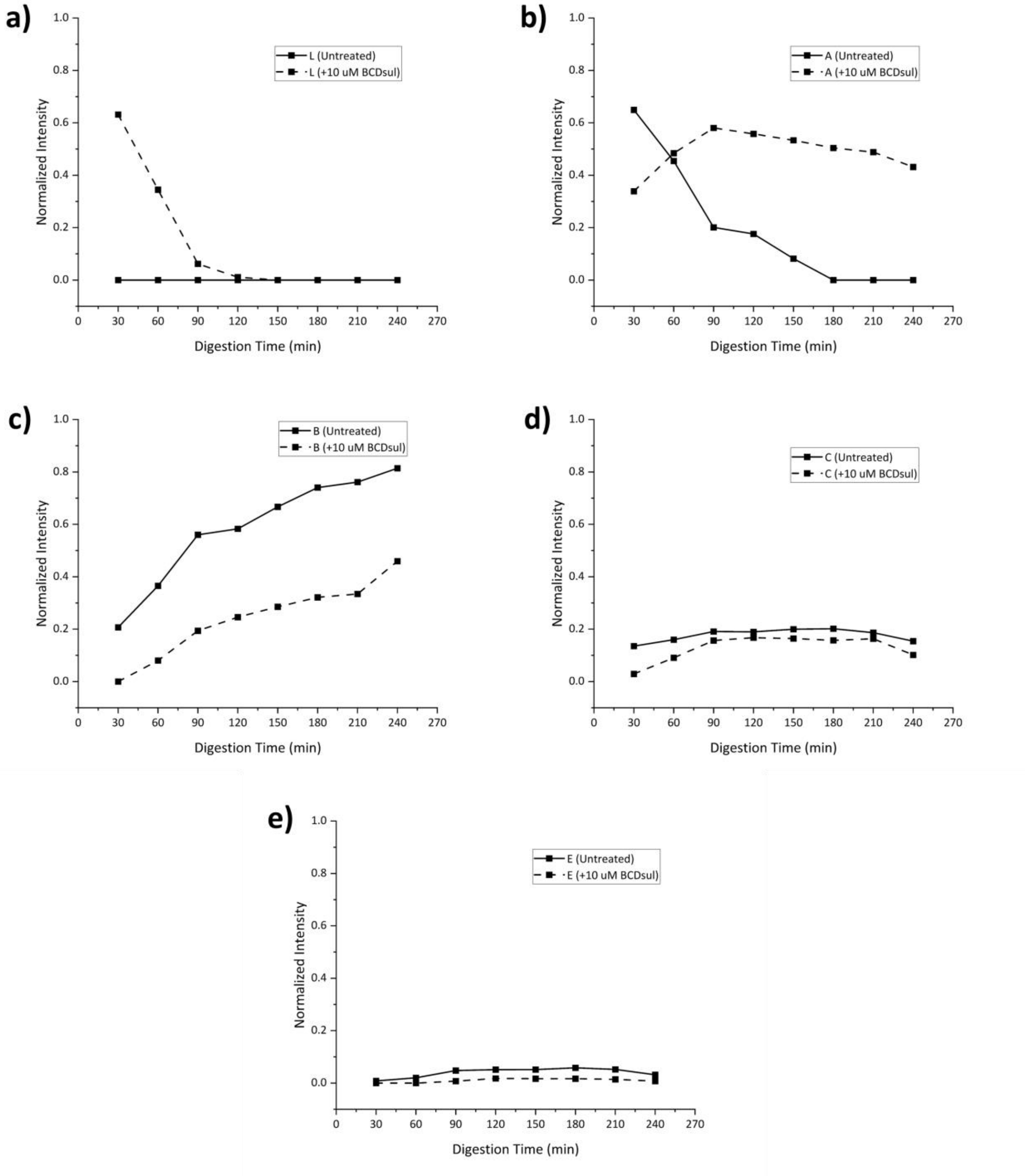

Figure S3: Time-course analysis of digestion for Fragments L, A, B, C, and E under untreated (solid line) and β-CDsul-treated (10 μM) (dashed line) conditions. Normalized signal intensity is plotted as a function of digestion time (in minutes) for five distinct components labelled (a) L, (b) A, (c) B, (d) C, and (e) E.

Dear Editor,

Please find enclosed our resubmission for our manuscript titled “Restriction Endonuclease Selective Inhibition of β-Cyclodextrin Sulfate”, BSR-2025-0403

In the email from your editorial team, we were told:

“It appears that University of Strathclyde is not participating in an R&P agreement. Therefore an article publishing charge (APC) will be due for payment if your paper is accepted.”

However, we have checked again on your website, which lists the University of Strathclyde as having entered into a read and publish agreement with Bioscience Reports – could you advise please, as this did inform our decision to publish with your journal.

Kind regards,

Fraser Scott
